# Is it possible for type 2 diabetic patients with low level of copper to have better glycemic control under different apolipoprotein B levels?

**DOI:** 10.3389/fendo.2026.1764209

**Published:** 2026-02-26

**Authors:** Zongheng Wu, Shumin He, Feng Zhu, Qiulan Gan, Sumei Li

**Affiliations:** 1 The Graduate School of Fujian Medical University, Fuzhou, Fujian, China; 2 Department of Endocrinology, The First Hospital of Putian City, Putian, Fujian, China; 3 Medical Department, Putian University, Putian, Fujian, China

**Keywords:** apolipoprotein B, copper, effect modifier, glycated hemoglobin, type 2 diabetes

## Abstract

**Objective:**

Copper overload has been implicated in impaired *β*-cell function and insulin resistance through oxidative stress and inflammatory pathways. As a major lipoprotein component involved in lipid oxidation, apolipoprotein B (apoB) may influence copper-related metabolic effects. This study aimed to examine the association between whole blood copper concentration and glycemic control in patients with type 2 diabetes mellitus (T2DM), and to assess whether apoB levels influence this association.

**Methods:**

A total of 117 patients with T2DM (mean age 55.15 ± 10.70 years; 62.4% male) were included. Whole blood copper concentration was measured using inductively coupled plasma mass spectrometry. Associations between blood copper and glycemic indicators, including glycated hemoglobin (HbA1c) and fasting plasma glucose (FPG), were evaluated using multivariable linear regression models. Stratified and interaction analyses were performed according to apoB and other lipid-related parameters.

**Results:**

After adjustment for potential confounders, a significant interaction between blood copper and apoB was observed in relation to HbA1c (P for interaction< 0.001). Stratified analyses showed that higher blood copper concentration was significantly associated with higher HbA1c levels among patients with lower apoB levels below the study median, whereas no significant association was observed among those with higher apoB levels. Exploratory analyses further indicated that apoB also influenced the association between blood copper and FPG (P for interaction< 0.05), showing a consistent pattern.

**Conclusion:**

In patients with T2DM, a significant association between blood copper concentration and glycemic control was observed among individuals with lower apoB levels, whereas no such association was found among those with higher apoB levels. These findings suggest that apoB status may influence the relationship between blood copper and glycemic control and merit further investigation in longitudinal studies.

## 1 Introduction

Type 2 diabetes mellitus (T2DM), defined by multiple metabolic disturbances, is a chronic, non-communicable condition, and it now constitutes a pressing worldwide health concern (1). International Diabetes Federation projections indicate that the global diabetes prevalence is set to reach 10.0% by 2045, and T2DM is anticipated to account for approximately 90% of these cases (2). T2DM is characterized by hyperglycemia, which arises from a combination of insulin resistance and impaired *β*-cell function (3). Poor long-term glycemic control is a known risk factor for serious macro- and microvascular outcomes, such as stroke, heart disease, and renal failure (4). As an integral biomarker for assessing glycemic status and a primary efficacy endpoint in T2DM clinical trials, glycated hemoglobin (HbA1c) provides a measure of average blood glucose concentrations from the preceding 2–3 months (5).

Beyond traditional metabolic risk factors, increasing attention has been directed toward the role of trace elements in glucose homeostasis and the pathophysiology of type 2 diabetes mellitus. Trace elements are involved in multiple biological processes, including oxidative stress regulation, inflammation, and insulin signaling, suggesting that disturbances in trace element homeostasis may contribute to impaired glycemic control (6). Copper (Cu) is crucial for multiple metabolic functions in the body as an essential trace element (7, 8). Functioning as an enzymatic cofactor—for instance, in cytochrome c oxidase and copper-zinc superoxide dismutase (Cu/Zn-SOD)—copper is involved in vital physiological functions such as mitochondrial respiration, ATP production, and antioxidant defense (9–11). Dysregulation of copper homeostasis is closely associated with abnormal glucose metabolism. Animal studies have demonstrated that diabetic mice exhibit significantly higher levels of serum copper and reactive oxygen species (ROS) compared to non-diabetic controls. Treatment with copper chelators not only reduced copper and ROS levels but also improved glucose intolerance and lowered serum triglycerides (12). Another study found disrupted copper homeostasis in diabetic rats, evidenced by reduced Cu/Zn-SOD activity. This implicates copper in the development of diabetes, potentially through mechanisms involving oxidative damage and inflammation (13). Clinical studies further indicate that circulating copper levels are significantly elevated in populations with diabetes relative to their healthy counterparts (14), and numerous studies have shown that blood copper levels are closely associated with the risk of diabetes (15–19). Excess copper in the body can promote oxidative stress and inflammation, thereby impairing pancreatic *β*-cell function, exacerbating insulin resistance, and ultimately contributing to poor glycemic control in T2DM patients (12, 20). However, existing findings on the relationship between blood copper and glycemic indicators are inconsistent: while most support a positive correlation (21–26), some report an inverse correlation (27) or no significant association (18), suggesting the potential presence of unrecognized effect modifiers.

Among lipid-related markers, apolipoprotein B (apoB) reflects the total number of atherogenic lipoprotein particles and has been suggested to be a more informative indicator of cardiometabolic risk than conventional lipid measures such as low-density lipoprotein cholesterol (LDL-C), which primarily quantify cholesterol content rather than particle number (28, 29). Because apoB captures the overall burden of circulating atherogenic lipoproteins, it may better reflect the lipid-related metabolic context relevant to the present study. Therefore, apoB was prioritized as a potential effect modifier in the association between whole blood copper concentration and glycemic control.

From a biological perspective, previous studies have suggested that copper can bind to ApoB-100 and be reduced within low-density lipoprotein (LDL) particles, thereby contributing to LDL oxidation (30). Oxidized LDL can further trigger inflammatory responses and oxidative stress (31), impairing *β*-cell function and leading to deteriorated glycemic control (32). In addition, multiple studies have reported positive correlations between blood copper levels and various lipid parameters, including apolipoprotein B (apoB) (33–35). Dyslipidemia itself is also closely associated with inflammation and oxidative stress (36, 37), which may further disrupt glycemic control in patients with T2DM (38).

However, it remains unclear whether apoB modifies the association between whole blood copper concentration and glycemic control in patients with type 2 diabetes mellitus. Therefore, the aim of this study was to investigate whether apoB modifies the association between whole blood copper concentration and glycemic control in patients with type 2 diabetes mellitus.

## 2 Materials and methods

### 2.1 Study population

A total of 117 patients with T2DM were enrolled in this retrospective cross-sectional investigation. All participants received care in the endocrinology outpatient or inpatient departments of the Department of Endocrinology, the First Hospital of Putian City, between January 1, 2025, and June 1, 2025. The study protocol received approval from the Ethics Committee of the First Hospital of Putian City, Fujian Province, China, and all procedures adhered to the tenets of the Declaration of Helsinki. Participants aged between 30 and 80 years were included in the study to focus on the typical adult T2DM population and to reduce potential confounding related to early-onset diabetes and advanced age–related comorbidities. The diagnosis of T2DM was based on the American Diabetes Association (ADA) diagnostic criteria (American Diabetes Association, 2024), requiring a fasting plasma glucose (FPG) ≥ 7.0 mmol/L and/or HbA1c ≥6.5% (39). All diagnoses were verified through a review of the electronic medical record system.

The study excluded individuals with: 1) severe infections or acute diabetic hyperglycemic states (such as ketoacidosis or hyperosmolar state); 2) autoimmune diseases; 3) severe dysfunction or insufficiency of vital organs, including the heart, liver, kidneys, or brain. Specifically, individuals with an estimated glomerular filtration rate (eGFR) ≤ 30 mL/min/1.73 m² were excluded. Informed consent was acquired from all subjects before their inclusion in the investigation. As this was a retrospective cross-sectional study, no longitudinal follow-up was performed. To minimize selection bias, all consecutive eligible patients meeting the inclusion and exclusion criteria during the study period were included.

### 2.2 Data collection

Data on demographic characteristics, clinical diagnoses, diabetes duration, use of antihypertensive drugs, and laboratory measurements were retrospectively extracted from the hospital electronic medical record system in July 2025, after completion of the study period. Lifestyle information, including smoking status and alcohol consumption, was obtained using structured questionnaires administered once at the time of the clinical visit by trained research staff, with relevant information cross-checked against electronic medical records. Body mass index (BMI) was calculated as weight in kilograms divided by height in meters squared (kg/m²). Although questionnaires were administered during routine clinical care, data extraction and analysis were performed retrospectively. Participants with missing data in key variables were excluded at the screening stage; therefore, the final analytical sample comprised 117 individuals with complete data on all variables included in the primary analyses.

Information on lipid-lowering therapy was extracted from electronic medical records when available. Patients receiving lipid-lowering treatment were included in the study to reflect real-world clinical conditions. Lipid parameters, including apoB and triglycerides, were analyzed using the measured values obtained at the time of assessment, reflecting the participants’ actual lipid status under routine clinical management. Data on dietary interventions and antioxidant supplementation were not systematically available and therefore were not included in the analysis.

### 2.3 Biochemical assays

Measurement of whole blood copper concentration was performed using inductively coupled plasma mass spectrometry (ICP-MS; Agilent 7850, USA) at a certified third-party clinical laboratory (ADICON Clinical Laboratory, China). Fasting venous whole blood samples were collected in trace-element–free EDTA anticoagulant tubes. Sample preparation, digestion, and ICP-MS measurements were conducted according to validated standard operating procedures routinely used for clinical trace element assessment. Calibration, internal standardization, and quality control procedures were implemented in accordance with established clinical laboratory quality assurance standards. Samples with visible hemolysis were excluded as part of routine laboratory quality control.

ICP-MS is a well-established analytical technique for trace element quantification in biological specimens and has been widely applied for measuring copper in whole blood and plasma (40, 41). The analytical performance of the ICP-MS method, including limits of detection, precision, and accuracy, has been previously described in the literature for clinical copper determination. In the present study, all reported copper concentrations provided by the laboratory were within the detectable range of the assay, and no values were reported as below the limit of detection.

FPG and fasting lipid parameters, including TC, TG, HDL-C, LDL-C, apoA, and apoB were measured in serum samples obtained from fasting venous blood using a Polaris c2000 automated biochemical analyzer (KHB, Shanghai, China). Glycated hemoglobin (HbA1c) was assessed in whole blood by high-performance liquid chromatography (HPLC) using a YH-60 analyzer (Hunan Yonghe-Sun Biotechnology Co., Ltd., China).

### 2.4 Statistical analysis

Statistical analysis was performed using SPSS Statistics (version 27) and R software (version 4.5.1). Normality of continuous variables was assessed using the Shapiro–Wilk test in combination with visual inspection of histograms and Q–Q plots. Continuous variables were summarized as mean ± standard deviation for normally distributed data or median (25th and 75th percentiles) for non-normally distributed data, while categorical variables were presented as number (percentage).

Baseline characteristics were compared between apoB-stratified groups using the independent-samples t-test for normally distributed continuous variables, the Mann–Whitney U test for non-normally distributed variables, and the chi-square test or Fisher’s exact test for categorical variables, as appropriate.

For stratified analyses, apoB was dichotomized at the median value (0.93 g/L). This approach was adopted to ensure balanced subgroup sizes and stable estimation of potential effect modification, given the skewed distribution of apoB and the absence of a universally accepted clinical cut-off specifically applicable to effect modification analyses in a cross-sectional context.

Spearman correlation analysis was initially conducted to explore pairwise associations among key variables. Multivariable linear regression models were then used to examine the association between whole blood copper concentration and HbA1c and to assess potential effect modification by lipid-related parameters and other factors. Two models were constructed: Model 1 was unadjusted, and Model 2 was adjusted for age, sex, BMI, patient type (outpatient/inpatient), and diabetes duration.

Whole blood copper concentration was treated as a continuous variable in all regression models to preserve statistical power and avoid information loss due to categorization. Effect modification by lipid parameters, age, sex, and BMI was evaluated using interaction tests and stratified analyses.

Potential nonlinear dose–response relationships between whole blood copper and HbA1c were assessed using restricted cubic spline (RCS) models with knots placed at the 10th, 50th, and 90th percentiles. The underlying assumptions of linear regression, including the normality of residuals, homoscedasticity, independence, and the absence of influential outliers, were verified. A two-sided P value< 0.05 was considered statistically significant for all analyses.

## 3 Results

### 3.1 Baseline characteristics of the study population

Baseline comparisons were descriptive in nature and intended to provide an overview of participant characteristics across apoB strata. Differences between apoB groups reflect characteristics associated with apoB stratification and were not interpreted for causal inference.

This study comprised a total of 117 participants. As shown in **Table 1**, the mean age of all participants was (55.15 ± 10.70) years, 62.4% were male, the mean BMI was (24.79 ± 3.11) kg/m², and 50.4% were inpatients. The concentrations of whole blood copper, HbA1c, FPG, and apoB showed non-normal distributions, with median (25th percentile, 75th percentile) values of 85.79 (78.17, 93.42) μg/dL, 8.00 (6.80, 10.65) %, 7.65 (6.52, 12.45) mmol/L, and 0.93 (0.76, 1.08) g/L, respectively.

**Table 1:** Participant characteristics (N = 117).

Variable	Total (N = 117)	Low apoB (N = 58)	High apoB (N = 59)	P value
**Age, years**	55.15 ± 10.70	56.43 ± 10.83	53.88 ± 10.51	0.199
**Male, n (%)**	73 (62.4)	35 (60.3)	38 (64.4)	0.650
**BMI, kg/m^2^**	24.79 ± 3.11	24.26 ± 2.95	25.31 ± 3.21	0.067
**Smoking, n (%)**				0.500
Yes	33 (28.2)	18 (31.0)	15 (25.4)	
No	84 (71.8)	40 (69.0)	44 (74.6)	
**Drinking, n (%)**				0.116
Yes	34 (29.1)	13 (22.4)	21 (35.6)	
No	83 (70.9)	45 (77.6)	38 (64.4)	
**Use of antihypertensive drugs, n (%)**	47 (40.2)	21 (43.1)	22 (37.3)	0.521
**Inpatient, n (%)**	59 (50.4)	24 (41.4)	35 (59.3)	0.052
**Diabetes Duration, years**	4.0 (1.0, 10.0)	5.0 (1.0, 10.0)	4.0 (0.4, 8.0)	0.237
**FPG, mmol/L**	7.65 (6.52, 12.45)	7.08 (6.01, 8.07)	11.06 (7.21, 13.42)	***<*0.001**
**HbA1c, %**	8.00 (6.80, 10.65)	7.45 (6.20, 8.70)	9.50 (7.20, 11.30)	***<*0.001**
**TG, mmol/L**	1.39 (0.91, 2.34)	1.17 (0.72, 2.09)	1.69 (1.22, 2.48)	**0.003**
**TC, mmol/L**	4.94 (4.17, 5.85)	4.17 (3.45, 4.78)	5.79 (5.18, 6.51)	***<*0.001**
**HDL-C, mmol/L**	1.18 (1.04, 1.45)	1.12 (0.91, 1.32)	1.29 (1.11, 1.52)	***<*0.001**
**LDL-C, mmol/L**	2.92 (2.21, 3.73)	2.24 (1.79, 2.70)	3.71 (3.14, 3.98)	***<*0.001**
**apoA, g/L**	1.17 ± 0.24	1.09 ± 0.21	1.25 ± 0.25	***<*0.001**
**apoB, g/L**	0.93 (0.76, 1.08)	0.76 (0.68, 0.86)	1.07 (0.99, 1.21)	***<*0.001**
**Whole blood copper concentration, *µ*g/dL**	85.79 (78.17, 93.42)	83.57 (77.53, 89.61)	87.06 (79.44, 95.33)	0.101

Bold text emphasizes statistically significant values. Values are expressed as mean ± standard deviation (SD) for normally distributed continuous variables, median (25th, 75th percentiles) for skewed continuous variables, and number (percentage) for categorical variables. P values are for comparisons between the Low and High apoB groups.

BMI, body mass index; FPG, fasting plasma glucose; HbA1c, glycated hemoglobin; TG, triglycerides; TC, total cholesterol; HDL-C, high-density lipoprotein cholesterol; LDL-C, low-density lipoprotein cholesterol; apoA, apolipoprotein A-I; apoB, apolipoprotein B.

Compared with the low apoB group, the high apoB group showed significantly higher blood glucose and lipid indicators (P< 0.05), while no statistically significant differences were observed between the two groups in baseline characteristics such as age, sex, body mass index, diabetes duration, and whole blood copper concentration (P *>* 0.05).

### 3.2 Association between whole blood copper concentration and HbA1c

Preliminary Spearman correlation demonstrated a weak but statistically significant positive association between whole blood copper concentration and HbA1c levels (r = 0.280, p = 0.002). To further investigate the independent effect of copper on HbA1c, we constructed multiple linear regression models (**Table 2**). In Model 1, which was unadjusted for any covariates, whole blood copper concentration showed a significant positive association with HbA1c levels (B = 0.057, 95% CI: 0.021 to 0.093, p = 0.002). However, after further adjustment for potential confounders including sex, age, BMI, diabetes duration, and patient type in Model 2, this association was no longer statistically significant (B = 0.023, 95% CI: -0.008 to 0.054, p = 0.148). In exploratory analyses, no significant association was observed between whole blood copper and FPG in either Model 1 or Model 2 (see **Table 2**, FPG section).

**Table 2:** Association between whole blood copper concentration and glycemic levels in 117 patients with type 2 diabetes.

Variable	Model 1		Model 2	
	*β* (95% CI)	P-value	*β* (95% CI)	P-value
HbA1c (%)				
Whole blood copper (*µ*g/dL)	0.057 (0.021, 0.093)	**0.002**	0.023 (-0.008, 0.054)	0.148
FPG (mmol/L)				
Whole blood copper (*µ*g/dL)	0.057 (-0.002, 0.116)	0.058	0.006 (-0.046, 0.058)	0.820

Bold text emphasizes statistically significant values. Data are presented as the unstandardized *β* coefficient with 95% confidence interval (95% CI), estimated by linear regression models. Model 1 is the crude model. Model 2 was adjusted for potential confounders, including sex, age, body mass index, duration of diabetes, and patient type (inpatient/outpatient).

### 3.3 Interaction analysis of blood copper and HbA1c stratified by key modifiers

**Figure 1:**
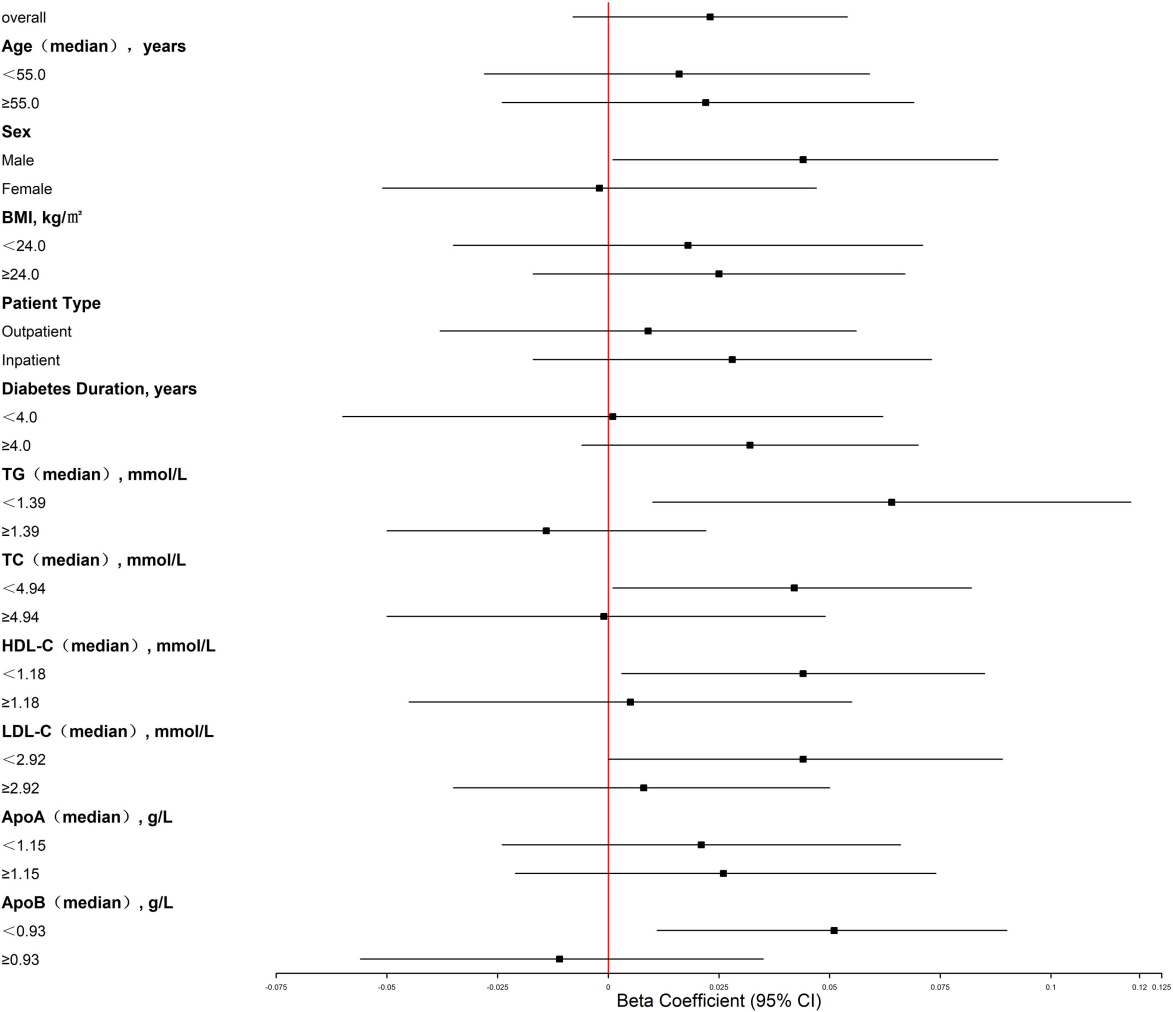
Forest plot for copper and HbA1c association in type 2 diabetes. Associations are beta coefficients with 95% CI from models adjusted for age, sex, BMI, diabetes duration, and patient type (N = 117). Square size represents point estimate; horizontal lines are 95% CIs. Vertical dashed line indicates null effect. Significant interactions with apoB (P-interaction< 0.001) and TG (P-interaction = 0.020). Positive association only in subgroups with apoB< 0.93 g/L or TG< 1.39 mmol/L. BMI, body mass index; TG, triglycerides; TC, total cholesterol; HDL-C, high-density lipoprotein cholesterol; LDL-C, low-density lipoprotein cholesterol; apoA, apolipoprotein A-I; apoB, apolipoprotein B.

A significant interaction was observed for serum apoB concentration, modifying the relationship of whole blood copper with HbA1c levels (P for interaction< 0.001; **Table 3**). Stratified analysis demonstrated a significant positive link between blood copper and HbA1c in individuals with low apoB levels (*<* 0.93 g/L) (B = 0.051; 95% CI: 0.011, 0.090; p = 0.013), conversely, no such significant association was detected in the high apoB subgroup (≥0.93 g/L) (B = -0.011; 95% CI: -0.056, 0.035; p = 0.639) (as shown in **Figure 1**). Furthermore, we systematically evaluated potential effect modification by a range of demographic, clinical, and metabolic parameters, including age, sex, BMI, patient type (inpatient or outpatient), diabetes duration, TG, TC, HDL-C, LDL-C, apoA, and apoB. Among all variables tested, only the interaction between TG and blood copper reached statistical significance (P for interaction = 0.020). The stratified pattern for TG was consistent with that of apoB: a positive association was observed in the low TG group (*<* 1.39 mmol/L) (B = 0.064; 95% CI: 0.010, 0.118; p = 0.021), in contrast, the high TG group showed no significant link (≥1.39 mmol/L) (B = -0.014; 95% CI: -0.050, 0.022; p = 0.442) (**Figure 1**). This implies that TG levels also moderate the relationship between copper and HbA1c to some extent. Detailed results are presented in **Table 3**.

**Table 3:** Stratified analysis and effect modification of the copper–HbA1c association in T2DM.

Variable	N	Blood copper (*µ*g/dL)	*β* (95% CI)	P-value	P-interaction
**Age (median), years**					0.508
*<* 55.0	55	84.0 (77.5, 88.3)	0.016 (−0.028, 0.059)	0.474	
≥55.0	62	88.3 (78.8, 94.8)	0.022 (−0.024, 0.069)	0.344	
**Sex**					0.281
Male	73	84.0 (76.6, 89.6)	0.044 (0.001, 0.088)	0.488	
Female	44	88.3 (80.7, 94.7)	−0.002 (−0.051, 0.047)	0.945	
**BMI, kg/m^2^**					0.424
*<* 24.0	46	84.8 (76.3, 92.9)	0.018 (−0.035, 0.071)	0.469	
≥24.0	71	85.8 (78.8, 94.1)	0.025 (−0.017, 0.067)	0.241	
**Patient type**					0.526
Outpatient	58	81.3 (77.4, 88.4)	0.009 (−0.038, 0.056)	0.706	
Inpatient	59	88.3 (82.0, 97.2)	0.028 (−0.017, 0.073)	0.225	
**Diabetes duration, years**					0.216
*<* 4.0	53	84.5 (78.2, 89.0)	0.001 (−0.060, 0.062)	0.973	
≥4.0	64	87.0 (78.1, 95.2)	0.032 (−0.006, 0.070)	0.095	
**TG (median), mmol/L**					**0.020**
*<* 1.39	58	83.3 (77.2, 90.0)	0.064 (0.010, 0.118)	**0.021**	
≥1.39	59	87.1 (79.4, 94.7)	−0.014 (−0.050, 0.022)	0.442	
**TC (median), mmol/L**					0.130
*<* 4.94	58	84.3 (77.2, 89.6)	0.042 (0.001, 0.082)	**0.043**	
≥4.94	59	87.1 (79.4, 94.7)	−0.001 (−0.050, 0.049)	0.978	
**HDL-C (median), mmol/L**					0.910
*<* 1.18	58	86.1 (78.0, 93.7)	0.044 (0.003, 0.085)	**0.038**	
≥1.18	59	84.5 (78.2, 93.4)	0.005 (−0.045, 0.055)	0.847	
**LDL-C (median), mmol/L**					0.137
*<* 2.92	57	85.8 (78.1, 92.0)	0.044 (0.000, 0.089)	0.051	
≥2.92	60	85.8 (78.3, 94.5)	0.008 (−0.035, 0.050)	0.725	
**ApoA (median), g/L**					0.460
*<* 1.15	57	85.8 (77.5, 94.1)	0.021 (−0.024, 0.066)	0.362	
≥1.15	60	85.8 (78.7, 93.3)	0.026 (−0.021, 0.074)	0.265	
**ApoB (median), g/L**					***<*0.001**
*<* 0.93	58	83.6 (77.5, 89.6)	0.051 (0.011, 0.090)	**0.013**	
≥0.93	59	87.1 (79.4, 95.3)	−0.001 (−0.056, 0.035)	0.639	

Bold text emphasizes statistically significant values. Data are presented as unstandardized *β* coefficients with 95% confidence intervals (95% CI), estimated from multivariable linear regression models. Adjusted confounders include age, sex, BMI, patient type (inpatient/outpatient), and diabetes duration.

HbA1c, Glycated hemoglobin; T2DM, Type 2 diabetes mellitus; BMI, body mass index; TG, triglycerides; TC, total cholesterol; HDL-C, high-density lipoprotein cholesterol; LDL-C, low-density lipoprotein cholesterol; apoA, apolipoprotein A-I; apoB, apolipoprotein B.

### 3.4 Exploratory analysis of the association between whole blood copper and FPG

Although the main effect of whole blood copper on FPG was not significant in either the unadjusted or adjusted models (Model 1: B = 0.057; 95% CI: -0.002, 0.116; p = 0.058; Model 2: B = 0.006; 95% CI: -0.046, 0.058; p = 0.820) (see **Table 2**, FPG section), we proceeded with pre-specified interaction analyses to further explore potential effect specificity. Interestingly, we found that apoB levels also significantly modified the association between whole blood copper and FPG (P for interaction = 0.012). Although the stratified analysis, likely owing to reduced statistical power, yielded non-significant p-values for individual subgroups, the direction and pattern of effects were consistent with the primary analysis using HbA1c as the outcome: a positive effect was observed in the low apoB group (*<* 0.93 g/L) (B = 0.038, p = 0.260), while a negative effect was found in the high apoB group (≥ 0.93 g/L) (B = -0.050, p = 0.165). This finding further supports apoB as a key effect modifier for the copper-glucose metabolism relationship.

### 3.5 Restricted cubic spline analysis of whole blood copper and HbA1c

**Figure 2:**
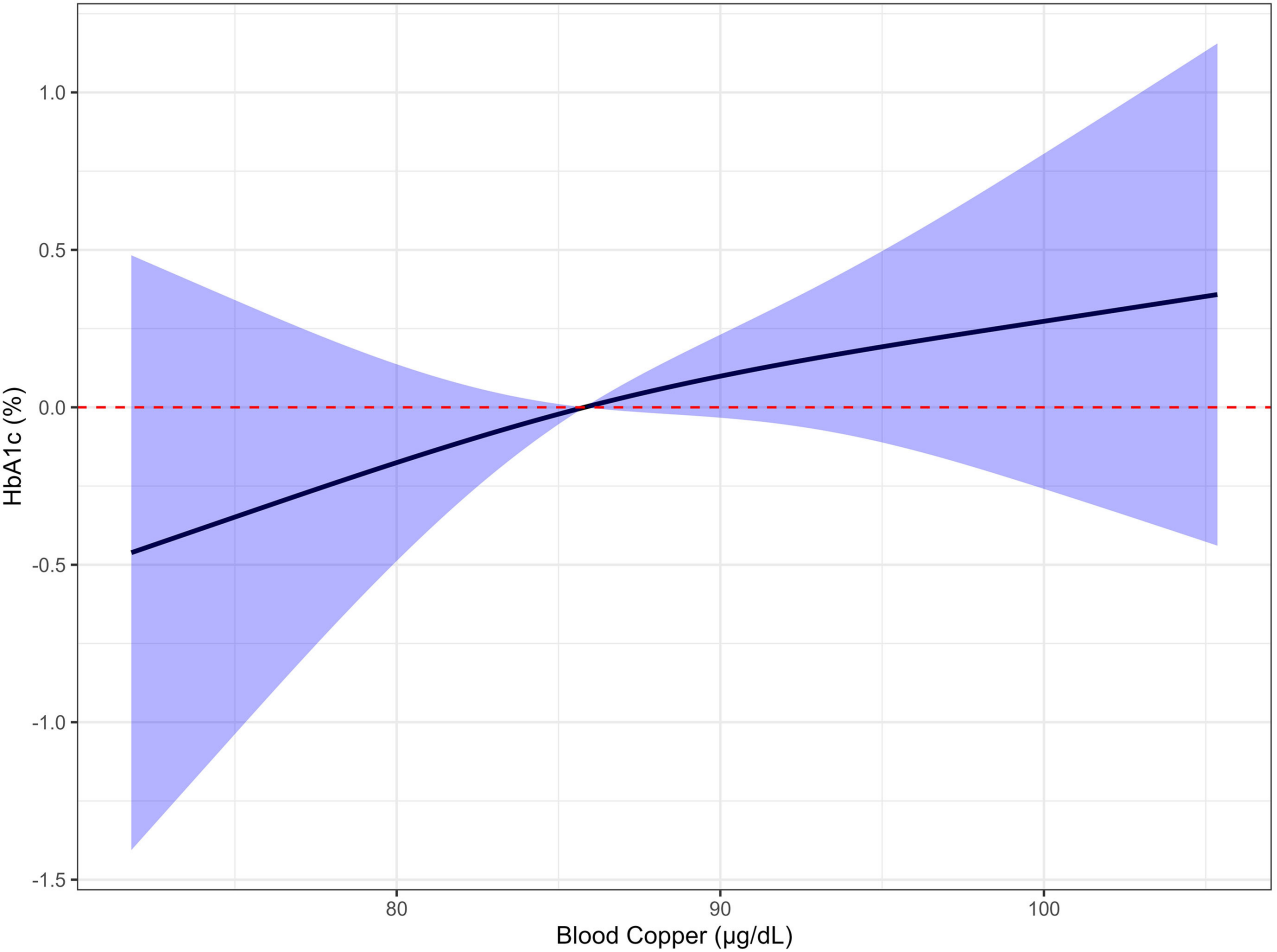
Restricted cubic spline analysis of blood copper and HbA1c in T2DM. Dose-response relationship using restricted cubic spline regression (3 knots at 10th, 50th, 90th percentiles). Adjusted for sex, age, BMI, patient type, and diabetes duration (N = 117). Solid curve: predicted HbA1c; blue shaded area: 95% CI; red dashed line: reference level. No significant nonlinear relationship (P-overall = 0.332, P-nonlinear = 0.737). HbA1c, glycated hemoglobin; T2DM, Type 2 diabetes mellitus; BMI, body mass index.

The relationship between whole blood copper concentration and HbA1c was analyzed using restricted cubic splines. Upon adjustment for key covariates including sex, age, BMI, patient type, and diabetes duration, the ANOVA model identified no significant departure from linearity in the association of whole blood copper with HbA1c (P-overall = 0.332, P-nonlinear = 0.737) (see **Figure 2**).

## 4 Discussion

In this study, we observed that whole blood copper concentration was not significantly associated with glycemic indices in the overall population of patients with T2DM. However, stratified analysis revealed that apoB levels significantly altered the relationship between blood copper and blood glucose, with a strong positive association observed only among individuals with lower apoB levels (**Table 3**).

The observed inconsistency in previous studies may, in part, reflect differences in lipid-related metabolic context. ApoB represents the burden of circulating atherogenic lipoprotein particles and is involved in copper-catalyzed LDL oxidation, which can amplify oxidative stress (28, 30). Therefore, variation in apoB levels may influence the association between blood copper concentration and glycemic regulation.

Based on the findings of this study, a biologically plausible explanation for the observed effect modification may relate to differences in the lipid-related metabolic context across apoB levels. Higher apoB concentrations reflect a greater burden of circulating atherogenic lipoprotein particles. Alterations in lipid metabolism and lipid-related indices have been associated with glycemic regulation in patients with type 2 diabetes mellitus in previous studies (38, 42). In this context, the association between blood copper concentration and glycemic control may differ according to apoB status, becoming more apparent in individuals with lower apoB levels. This context-dependent association may partly explain why the copper–HbA1c relationship was not evident in the overall analysis but emerged in the stratified analysis.

The modifying effect observed for TG is consistent with the findings for apoB and supports the involvement of lipid-related metabolic pathways in shaping the association between blood copper and glycemic control. TG-rich lipoproteins, such as very-low-density lipoproteins, contain apolipoprotein B and are susceptible to copper-induced oxidative modification (43), suggesting that the TG interaction likely reflects a related lipid metabolic context rather than an independent pathway distinct from apoB.

Notably, exploratory analyses using FPG as an outcome demonstrated a similar pattern of apoB-dependent modification, indicating that this phenomenon is not limited to HbA1c but may extend to broader aspects of glucose homeostasis. Together, these findings suggest that the lipid metabolic environment, reflected by apoB and related lipid parameters such as TG, represents an important background context influencing the copper–glycemia relationship.

As far as we are aware, this is the first study to report a modifying role of apoB in the association between blood copper concentration and HbA1c. Our findings may help to contextualize previously inconsistent reports on copper and glycemic control. Prior evidence suggesting that HDL modifies the copper–diabetes relationship (44) further supports the notion that the relationship between copper and glucose metabolism is strongly dependent on the overall lipid milieu.

Several limitations should be acknowledged. First, the retrospective cross-sectional design precludes causal inference between whole blood copper concentration and glycemic control. Second, this was a single-center study with a relatively modest sample size, which may limit statistical power and generalizability. Therefore, the findings should be interpreted as hypothesis-generating and warrant confirmation in larger, prospective studies. Third, information on dietary intake, potential environmental exposure sources of copper, and antioxidant supplementation was not systematically available and could not be evaluated. In addition, the study population consisted exclusively of Chinese patients, which may limit the generalizability of the findings to other ethnic groups. Finally, zinc concentrations were not available in the present study, precluding assessment of the zinc-to-copper ratio, which has been suggested to be relevant to glycemic regulation in T2DM.

This study suggests that the association between blood copper concentration and glycemic control in patients with T2DM may differ according to apoB levels. The observed effect modification highlights the potential importance of lipid-related metabolic context when evaluating the relationship between trace elements and glucose metabolism. Although no causal or therapeutic conclusions can be drawn from this cross-sectional analysis, our findings provide a rationale for further prospective and interventional studies to investigate whether combined modulation of copper status and lipid metabolism may have implications for glycemic regulation in specific metabolic subgroups of patients with T2DM.

## 5 Conclusions

In patients with type 2 diabetes mellitus, whole blood copper concentration was not associated with glycemic control in the overall population. However, apoB levels modified this association: among individuals with lower apoB levels, lower blood copper concentration was associated with better glycemic control, whereas no significant association was observed in those with higher apoB levels. These findings indicate that the relationship between blood copper and glycemic regulation in T2DM may depend on the underlying lipid metabolic context. Given the cross-sectional design, these results are hypothesis-generating and warrant confirmation in future longitudinal and interventional studies.

## Data Availability

The raw data supporting the conclusions of this article will be made available by the authors, without undue reservation.
